# Higher temperatures are associated with increased risk of police violence: A nationwide county-level study in the United States, 2013–2024

**DOI:** 10.1371/journal.pone.0345523

**Published:** 2026-03-20

**Authors:** Jiacheng Zou, Kun Hou, Xia Xu, Zhen Wang

**Affiliations:** 1 School of Remote Sensing and Geomatics Engineering, Nanjing University of Information Science and Technology, Nanjing, China; 2 Jiangsu Province Hydrology and Water Resources Investigation Bureau, Nanjing, China; 3 National Geomatics Center of China, Beijing, China; Nanyang Technological University, SINGAPORE

## Abstract

Ambient temperature has been demonstrated to be associated with a variety of violent or conflict events. However, few studies have so far linked temperature to the risk of police violence, where the quantitative estimate of the temperature effect is still unclear. In this study, we comprehensively explore the relationship between temperature variations and police violence risk based on a series of panel regression models with high-dimensional fixed effects in the United States. The results indicate a generally positive association between temperature and the police violence, with higher temperatures corresponding to elevated risks. The heterogeneity analysis exhibits that lower levels of precipitation and larger population sizes are associated with increased risks of police violence. Specifically, under the conditions of less than 50 mm precipitation and a population of larger than 5 million, each 1°C rise in monthly average temperature is associated with an increased death rate of 2.06 (95% CI: 0.92–3.20) and 2.01 (95% CI: 1.08–2.93), respectively. The temperature effect on police violence risk presents notable spatiotemporal variation, with elevated risks observed in certain states with experiencing high temperature and particularly during the year of 2024. Our research projects that by the year 2050, under the highest greenhouse gas emissions scenario of SSP5–8.5, the cumulative additional deaths from police violence in the United States due to expected temperature change would achieve 479 (95% CI: 183–836). Given the profound and widespread societal impact of deaths related to police violence, these projected additional deaths may pose further challenges to public health and social stability in the United States. Our research reveals the linkage between temperature variation and the risk of police violence, highlighting the urgent need for targeted intervention strategies in the practices of police law enforcement, particularly under the high-temperature environmental conditions.

## 1. Introduction

The trend of sustained climate warming is occurring on a global scale at an unpredictable rate [1]. Global warming is accelerating much faster than previously estimated, with warming over the past 15 years almost twice as fast as in the past 40 years [2]. Since around 1990, there have been clear signs of an increase in the warming rate, with surface temperature increases being more pronounced in recent La Niña-affected years [3]. Global average surface temperatures are 1.43°C above pre-industrial levels in 2023, which exceeds the best estimate of human-induced warming of 1.31°C [4]. The following year of 2024 became the hottest year globally on record, the first calendar year to exceed 1.5°C [5]. High temperature exposure caused by unstoppable and rising temperatures has a broad and significant adverse impact on a wide range of health outcomes and public safety events, ranging from mortality [6,7], suicide [8,9], multiple diseases [10–13] to various types of violence or crime [14–17].

Researchers from different regions or countries around the world have turned their attention to the associations between heat exposure and violent or criminal events, among which the quantitative effect of high temperature on the increased risk has been confirmed. High temperatures were significantly associated with crime, violence, or both, with a 10°C increase in short-term average temperature exposure associated with a 9% increase in the risk of violent crime [14]. Ambient temperature was found to account for 10% of the variation in violent crime rates in Finland, suggesting that a 2°C increase in mean temperature would lead to a more than 3% increase in violent crime rates in non-tropical and non-subtropical regions [18]. Exposure to high temperatures was found to be significantly associated with the occurrence of five types of violent incidents in the Greater Middle East, and short-term high temperatures may increase the risk of most forms of violent conflict [19]. Research focusing on the relationship between extreme temperatures and violent mortality in Russia shows that extreme heat increases violent mortality, whereas extreme cold has no effect [17]. A non-linear relationship was demonstrated between daily summer temperatures and the number of violent crimes, with assaults and domestic violence increasing significantly as temperatures rise [20].

Several studies have also examined the linkage between heat exposure and risk of violence or crime in the United States. Average daily high temperatures are associated with violent crime, with violent crime rates under high temperatures being 1.03 times higher than that under moderate temperatures, which are also higher than violent crime rates under cold temperatures [16]. The frequency and severity of climate-driven extreme heat events promote a positive correlation between heat and violence in U.S. cities [21]. High temperatures were found to exert a positive effect on both domestic and non-domestic violence risk in Chicago, whereas the effect of low temperatures was less pronounced [22]. The risk of gun violence was confirmed to increase plausible monotonically with rising temperatures, with higher temperatures corresponding to higher relative risk, and even moderately hot temperatures were associated with a higher risk of gun violence in 100 large U.S. cities [23]. The studies of samples in these regions and across the United States have demonstrated a clear association between heat exposure and increased risk of violence and crime.

The burden of lethal police violence is an urgent public health crisis facing the United States [24–27]. There is growing evidence that violent deaths caused by police officers in the name or for the sake of law enforcement are important triggers for social unrest and large-scale public safety and health events, as evidenced by several high-profile police killings in recent history [25–27]. The Global Burden of Disease, Injury and Risk Factor Study (GBD) found that in 2019, the United States, with just 4% of the world’s population, accounted for 13.2% of the 8,770 deaths worldwide due to police violence [26]. Police violence is the leading cause of death for young men in the United States, with the risk peaking between ages 20 and 35 for both sexes and regardless of race and ethnicity [25]. Several vivid examples of fatal police violence over the past few years remind us that police violence must be treated as a severe public health threat [28]. Previous studies have provided solid and reliable evidence of the facilitative effects of high temperatures or additional heat exposure on violent or criminal incidents. However, the question of how the risk of police violence is affected by ambient temperatures is less well understood, and the quantified effects of heat-related risk remain unclear. The impact of environmental temperature exposure on the risk of police violence needs to be further elucidated.

Here, leveraging the nationwide data of police violence deaths, temperature and precipitation in the United States from January 1, 2013 to December 31, 2024, we address an important empirical gap by investigating the relationship between heat exposure and the risk of police violence, a topic that has been largely overlooked despite the increasing prevalence of police violence and the challenges posed by climate change. While previous studies have explored temperature and violent outcomes, research focusing specifically on policing remains limited. To the best of our knowledge, this is the first nationwide analysis to quantify the impact of temperature variations on police violence at the county level across the United States. We make a contribution by employing panel regression analysis with high-dimensional fixed effects, which allows us to control for unobserved heterogeneity across counties such as demographic composition, baseline crime levels, and policing practices, as well as nationwide shocks and seasonal patterns over time. This approach enhances the rigor and precision of our estimates and enables us to characterize the nonlinear relationship between temperature and the death rate of police violence, while also accounting for the displacement effect of temperature. Furthermore, we estimate the potential number of additional excess deaths attributable to future temperature increases under the highest greenhouse gas emission scenario of SSP5–8.5. Our findings not only advance the methodological and empirical literature on temperature-related public health and social outcomes but also sound the alarm for the emerging public health crisis of police violence in the United States under the context of climate change.

## 2. Materials and methods

### 2.1 Police violence data

We use the deaths of police violence to represent the police violence, which is derived from the website of Mapping Police Violence (MPV) (https://mappingpoliceviolence.org/) [29, 30]. As one of the most comprehensive databases of victims of police-related fatalities in the continental United States, the MPV dataset integrates information on deaths caused by police officers using batons, shootings, chokeholds, stun guns or other methods while on duty or off duty across the United States from 2013 to the present, including information involving race, age, gender, number of deaths, time of occurrence, and location of the incident (using the latitude and longitude in the report to locate it to the county level). We restricted our analysis to police violence incidents with complete and reliable information. The original dataset contained 14,307 records and was systematically processed for analysis. Administrative district codes were matched, and entries with missing information such as latitude, longitude, or incident address were removed. Blank values after successful matching were also addressed to prevent geographic misclassification or inaccurate case identification. In addition, cases categorized as vehicle or unknown were excluded prior to analysis. After these procedures, 13,381 records from January 1, 2013 to December 31, 2024 were retained as valid observations in this study, ensuring that the dataset accurately reflected the incidents of police violence and provided the reliable spatial information. S1 Fig illustrates the spatial distribution of police violence incidents across the United States.

### 2.2 Meteorological data

The meteorological data used in this study were derived from the climate monitoring database of the National Centers for Environmental Information of the National Oceanic and Atmospheric Administration (NOAA NCEI) (https://www.ncei.noaa.gov/) [31], which obtains raw data of meteorological observations from multiple meteorological monitoring stations across the United States. Its statistical information has high precision and accuracy, and the data has been centrally reported to the World Meteorological Organization simultaneously. The dataset was spatially interpolated using the inverse distance weighted (IDW) method to align the station data with the geographic center point of the county to generate temperature and precipitation data for each county. The meteorological data we collected spans from January 2013 to December 2024, including monthly average temperature (°C) and precipitation (mm), which are calculated by averaging the daily data of the NOAA sites.

### 2.3 The main regression model

We use a high-dimensional fixed effects model to explore the association between temperature variations and death rate of police violence. In this study, the death rate of police violence is defined as the number of deaths caused by police violence in a given county divided by the county’s population. This normalization allows us to control for population size, since larger counties naturally experience more incidents simply due to their population base, while smaller counties experience fewer. By adjusting for population, this rate provides a more accurate measure of the relative magnitude of police violence across counties. Fixed effects is a statistical method commonly used in panel data analysis, which controls for unobserved interference effects by introducing the individual or time-specific terms. We apply the population weights to account for the differences in county population size, cluster by the county for robust standard error estimation, and use the fixed effects to capture the time-varying factors at the state-year and county-month level, thereby separating the effects of temperature and precipitation on the death rate of police violence [32]. The main regression model constructed is shown in Eq. (1):


$$y_{imst}\;=\;f\;\left(\;{\text{temp}}_{ims\text{t}}\right)+\;\beta {\text{prec}}_{ims}\;+\;\delta_{\text{im}}+\alpha_{\text{st}}\;+\;\mu_{ims\text{t}}\;$$ (1)

where *i* indicates the county where the death incident of police violence occurred, *s* denotes the state to which the county belongs, *m* represents the month of the year, and *t* indexes the year. ${\text{y}}_{\text{imst}}$ represents the death rate of police violence in the month of a given county, which is calculated using the number of deaths from police violence that month divided by the county’s population. ${\text{temp}}_{\text{imst}}$ and ${\text{prec}}_{\text{imst}}$ represent the monthly average temperature and precipitation of that county, respectively. The function *f* represents the nonlinear relationship between temperature and death rate of police violence modeled using natural cubic splines of three nodes (nodes placed at 0°C, 10°C, and 20°C), higher-order polynomials, B-spline, and natural cubic curves of seven nodes (nodes evenly distributed between −17°C and 33°C), respectively. $\beta {\text{prec}}_{\text{imst}}$ is incorporated into the model in a linear manner to control for the precipitation effects, $\alpha_{\text{st}}$ indicates the state-year fixed effect to control for the annual trends within the state, $\delta_{\text{im}}$ represents the county-month fixed effect to accounts for other local factors may be associated with the deaths of police violence, such as seasonal variations in the monthly cycle across different counties, and $\mu_{\text{imst}}$ is the random error term. We use the Akaike information criterion (AIC) [22,33] to select the optimal fit, where the model of three nodes of the natural cubic spline corresponds to the lowest AIC value, and it is referred to as the best fit.

To further assess the quantitative effect of temperature on the death rate of police violence, we substitute the constant term γ for the function *f* to derive a linear model with fixed effects of the temperature variable [22,34], as shown in Eq. (2):


$$y_{imst}\;=\gamma \;(\;{\text{temp}}_{ims\text{t}})\;+\;\beta {\text{prec}}_{imst}\;+\;\delta_{\text{im}}+\alpha_{\text{st}}\;+\;\mu_{ims\text{t}}\;$$ (2)

where the results of the estimate of γ are considered to be the effect of a 1°C deviation from normal, or the effect of a 1°C increase in absolute temperature. The remaining variables and terms remain unchanged. To facilitate the interpretation and comparison of the results, we set the expected death rate at 10°C as the baseline level when drawing the temperature-risk response curve to visually exhibit the increase or decrease in death rate above or below this temperature [22,32]. We also superimposed a histogram of the temperature distribution in the graphical results to demonstrate the data density of each temperature range in the sample. All models were weighted regression using the *felm* function in the lfe package of the R software under the same fixed effect setting, and statistical tests were performed using robust standard errors clustered by county [32].

While monthly data struggle to accurately capture immediate responses to short-term fluctuations in temperature (such as day-to-day swings, which have been documented in previous studies), it is better able to reveal those shifts in impacts that can last for weeks and have been demonstrated in studies of weather-related violence [32,34]. In order to explore whether the impact of temperature on police violence risk shifts over time, we added the previous month’s temperature and the next month’s temperature as the additional exposure variables based on model (2) to assess the potential time-shift effect, which is shown in Eq. (3):


$$y_{imst}\;=\sum_{L=-\text{1}}^{\text{1}}(\gamma^{L}{Temp}_{is,(m-L)t}\;+\;\beta^{L}{Prec}_{is(m-L)t})\;+\;\delta_{\text{im}}+\alpha_{\text{st}}+\mu_{ims\text{t}}$$ (3)

where $\gamma^{\text{L}=\text{0}}$ represents the impact of the temperature of the current month, $\gamma^{\text{L}=\text{1}}$ represents the impact of the temperature of the previous month, and $\gamma^{\text{L}=-\text{1}}$ represents the impact of the temperature of the next month. By estimating the above model, we examine the impact of temperature in different time exposure windows on the death rate from police violence [32]. Similar to previous studies [7,34], the sum of the effects of previous month and current month of temperature were referenced as the overall effect for the death rate of police violence.

### 2.4 Heterogeneity analysis

To examine the heterogeneity of the effect of temperature on the death rate of police violence, we extended the baseline model to introduce the interaction terms to capture the heterogeneous responses [32,35], as shown in Eq. (4).


$$\;y_{imst}\;=\;\gamma_{\text{1}}{Temp}_{ismt}\;+\gamma_{\text{2}}({Temp}_{ismt}\;\times {\text{D}}_{\text{i}}{)+\;\beta_{\text{1}}{Prec}_{ismt}+\beta_{\text{2}}({Prec}_{ismt}\times {\text{D}}_{i})+\delta_{\text{im}}+\alpha }_{\text{st}}\;+\;\mu_{ims\text{t}}$$ (4)

where Di indicates that when the variable of the i_th_ region meets the specified conditions, the value is 1; otherwise, the value is 0. The coefficients of γ_2_ and β_2_ are calculated and reported, respectively, which are interpreted as the heterogeneous responses of temperature and precipitation to the risk of police violence under the situation of the variables of interest [32,35]. The introduction of interaction terms to estimate the heterogeneity may not fully reflect the adaptability of police violence to extreme temperatures. Therefore, we use a binned model to further investigate the heterogeneous response, where the samples are grouped according to monthly temperature (<10°C, 10–20°C, > 20°C), precipitation (<50mm, 50–100 mm, > 100mm) and population size (<1 million, 1–5 million, > 5 million). The linear effect of the model is estimated in each subgroup based on Eq. (3) to analyze the differences in temperature effects within the subgroups.

At the same time, in order to assess the specific effects of temperature within different year or state, we interact the variables of temperature and precipitation with the dummy variable of state or year on the basis of Eq. (4) (${Temp}_{ismt}$ and ${Prec}_{ismt}$ are multiplied by state and year, respectively), and present the interaction coefficients of each state or year separately to explore the quantitative effects of these variables at the state level in the spatial dimension and at the year level in the temporal dimension [32,35], as shown in Eqs. (5) – (6):


$$y_{imst}\;=\;\gamma_{\text{3}}{Temp}_{ismt}\;+\gamma_{\text{4}}({Temp}_{ismt}\;\times {\text{Y}}_{\text{t}}{)+\;\beta_{\text{3}}{Prec}_{ismt}+\beta_{\text{4}}({Prec}_{ismt}\times {\text{Y}}_{t})+\delta_{\text{im}}+\alpha }_{\text{st}}\;+\;\mu_{ims\text{t}}$$ (5)

$$y_{imst}\;=\;\gamma_{\text{5}}{Temp}_{ismt}\;+\gamma_{\text{6}}({Temp}_{ismt}\;\times {\text{P}}_{\text{s}}{)+\;\beta_{\text{5}}{Prec}_{ismt}+\beta_{\text{6}}({Prec}_{ismt}\times {\text{P}}_{s})+\delta_{\text{im}}+\alpha }_{\text{st}}\;+\;\mu_{ims\text{t}}$$ (6)

where Y_t_ and P_s_ denote the dummy variable of state and year, respectively. The interaction coefficients $\gamma_{\text{4}}$ and $\beta_{\text{4}}$ represent the estimated effects of temperature and precipitation in a specific year, respectively, reflecting the impact of temperature on the death rate of police violence in year *t*, and the differences between states can also be compared through the corresponding coefficients of $\gamma_{\text{6}}$ and $\beta_{\text{6}}$. This model is designed *t*o detect whether the effects of temperature and precipitation on the death rate of police violence change over time (2013–2024) and space (the 49 states of the continental United States). We estimate an alternative version of Eqs. (5) and (6), substituting the month and climate zone variables for the terms of year and state, to further respectively quantify the impact of temperature effects across different months and climate zones. The climate zone classification follows the nine climate consensus zones defined by NOAA for the contiguous United States [36,37].

### 2.5 Effects of future temperatures on police violence risk

To assess the potential impact of future climate changes on the deaths of police violence, we analyzed climate projections from the Coupled Model Intercomparison Project phase 6 (CMIP6) [38,39]. Specifically, we employed the projections under the high greenhouse gas emission scenario (Shared Socioeconomic Pathway 5–8.5, SSP5–8.5) emissions scenario, which assumes a continued substantial increase in greenhouse gas emissions through 2050, and our data were sourced from 30 global climate models that provide SSP5-8.5-based projections of mean temperature changes [32]. The processing of climate projection data followed a standardized approach across all these 30 models. Monthly temperature changes were estimated for each climate grid cell by calculating the difference between the mean projected temperatures for the late mid-century (2045–2050) and those for the baseline period (2013–2024). These model grids were then spatially aligned with the administrative units of the study (i.e., U.S. counties), and location-specific temperature changes were determined by averaging the values from overlapping grid cells, weighted by their respective contributions to each administrative unit. This methodology yielded the county-level estimates of expected temperature changes across the United States by 2050 [32].

We integrated localized climate projections with historical estimates of temperature effects on police violence-related death rate to quantify the potential percentage change in death rate due to warming by 2050 and the cumulative excess deaths over this period. The percentage change in death rate was derived by applying the historical temperature-death rate relationship while incorporating both current and lagged effects to account for the potential displacement to population-weighted temperature projections from 30 climate models spanning 2020–2050. The total excess deaths from police violence attributable to climate warming during this period were then calculated as follows:


$${\text{E}}_{\text{c}}\;=\;\sum_{\text{t}=\text{2020}}^{\text{T}=\text{2050}}{pop}_{ct}\times (\Delta {\text{T}}_{\text{ct}}\times \beta )$$ (7)

where *pop*_*ct*_ represents the predicted population of county *c* in year *t*, which is derived from the United Nations population projections [40]. β represents the estimated net change in death rate of police violence per 1°C warming (lagged plus current), and *ΔT*_*ct*_ accounts for the projected temperature rise for the corresponding county between 2020 and year *t*. We estimate β by applying Eq. (3) to the empirical data from 2013–2024. Previous research indicates that climate change will cause varying degrees of warming across different seasons [32,41]. Therefore, it is appropriate to apply the projected change in annual average temperature (*ΔT*_*ct*_) to the monthly temperature-death rate coefficient β. To account for the uncertainty, we employ a bootstrapping approach, resampling historical estimates of the death rate-temperature association 1,000 times with replacement. The resulting temperature sensitivity distribution is then integrated with each of the 30 climate model projections, yielding a total of 30,000 Monte Carlo potential projections [32,42].

### 2.6 Robustness analysis

To ensure that the results are robust to the model specification and data selection, we conducted several robustness tests. First, we estimate the main model using regression methods with alternative fitting functions and different specifications, including natural cubic spline with different degrees of freedom, higher-order polynomial, and B-spline, and find that the curves remain largely consistent (see Results), indicating that the results are not driven by the choice of functional form. Second, we expanded the range of the displacement effect from current one month to previous or following two months to assess whether our study includes the full length of the displacement effect, and the temperature effect on the death rate of police violence within each range was quantified, respectively. At the same time, we varied the combinations of confounding factors in the model, including the precipitation and the variables of state-year and county-month. These results confirm the reliability of our study.

## 3. Results

### 3.1 Nonlinear relationship between temperature and death rate of police violence

**Fig 1:**
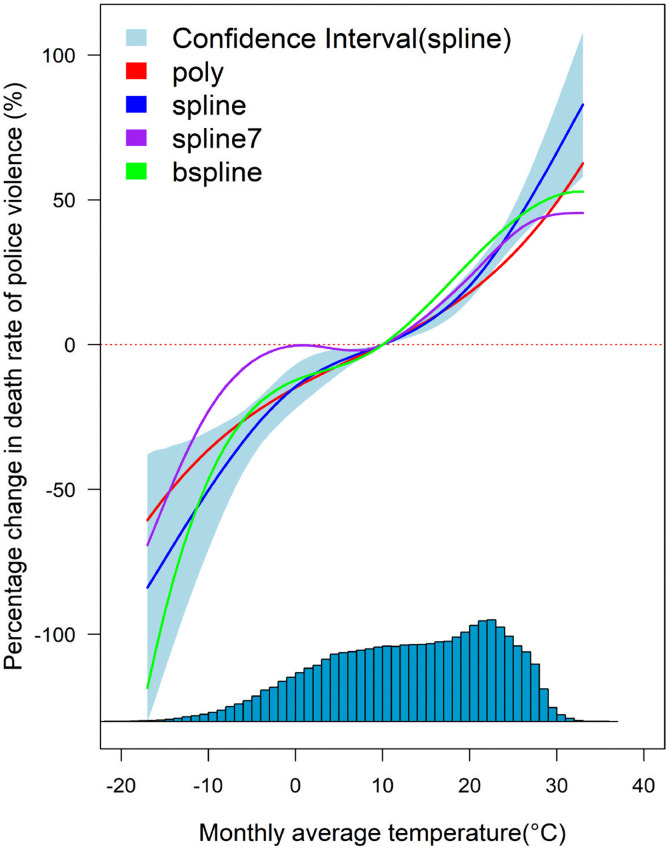
Nonlinear relationship between climate variation and death rate of police violence. The red, blue, purple, and green curves represent the fitting functions with respect to the high-order polynomial, natural cubic spline with three nodes, natural cubic curves with seven nodes, and B-spline, respectively. The light blue area indicates the confidence interval of the natural cubic spline with three nodes. The histogram above the horizontal axis presents the distribution frequency of the monthly average temperature.

Different fitting functions including high-order polynomial, natural cubic spline with three nodes, natural cubic spline with seven nodes, and B-spline were utilized to describe and characterize the nonlinear relationship between temperature variation and death rate of police violence (see Methods). The principle of the minimum AIC value of the Akaike information criterion is used to assess and choose the best fit [22,33], where the natural cubic splines with three nodes are validated and selected. Fig 1 illustrates the association between the variations in monthly mean temperature and the death rate of police violence, where a nonlinear exposure-response relationship is presented. The temperature-death rate curve generally demonstrates an upward trend, and the risk of police violence increases significantly as the average temperature of the month exceeds 20.3°C or is below −3.2°C. As monthly mean temperatures range from −3.2°C to 20.3°C, and the estimated effect of temperature on the death rate of police violence is relatively weak. The results also exhibit that the effect of temperature rise on death rate of police violence under high temperature conditions is slightly more pronounced than that of under low temperatures. Overall, we found that higher temperatures were positively associated with an increase in the risk of police violence, and this association was more pronounced at temperatures above 20.3°C or below −3.2°C (with a greater slope value for the upward curve). Therefore, in the subsequent studies, we used a linear panel regression high-dimensional fixed-effect model to estimate the quantitative effect of temperature variations within these ranges on the death rate of police violence.

### 3.2 Heterogeneity in the impact of temperature on police violence risk

**Fig 2:**
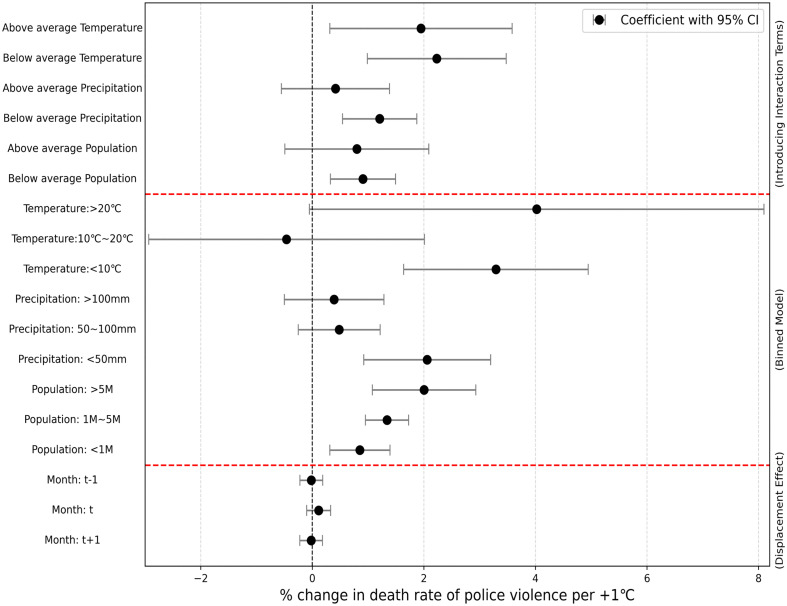
Temperature effects on the death rate of police violence across different subgroups. The upper, middle, and lower parts of the figure (divided by red dashed lines) represent the heterogeneity analysis with the introduction of interaction terms, the heterogeneity analysis of the binned models, and the displacement effects, respectively. The estimated effect of temperature is marked by the black solid circles, with grey lines illustrating their 95% confidence intervals.

We employ distinct approaches to explore the heterogeneity of temperature effects on the death rate of police violence, in order to fully reflect the adaptability of police violence risk to extreme temperatures and enable the purpose of mutual verification simultaneously. First, we introduce the interaction terms into the model to examine the differential performance of temperature effects across key variables of interest [32,35]. Second, we apply a binned strategy to further investigate the heterogeneous responses, grouping the sample according to the level of monthly temperature, precipitation, and population size. Within each defined subgroup, we independently estimate the temperature effect, allowing for a detailed analysis of heterogeneity in response across different conditions (see Methods). Fig 2 and Table 1 exhibit the effect of temperature effects on the death rate of police violence across different subgroups, where the introduction of interaction terms, grouping analysis of the binned model, and the displacement effects are presented separately. The results indicate that the effect of temperature on the death rate of police violence varies with multiple factors, and the heterogeneity under different background variables is confirmed. The temperature effect of 2.01 (95%CI: 1.08–2.93) in populous counties (> 5 million) is greater than that of 0.85 (95%CI: 0.31–1.39) in less populated counties (< 1 million). Precipitation also moderates the temperature impact, and the temperature effect of 2.06 (95%CI: 0.92–3.20) was more pronounced with less precipitation (<50 mm) than 0.39 (95%CI: −0.50–1.28) with higher precipitation (>100 mm). Both the interaction terms and the binned analysis indicate that the effect of temperature on death rate of police violence is greater under high temperature conditions than that of under low temperatures, which aligns with Fig 1 and the findings of prior research [32]. This is mainly because temperature variation is more sensitive to the effect of police violence under higher temperature conditions. We found that the effect of the previous month’s temperature (t-1) was negative of −0.02 (95% CI: −0.22–0.18), the temperature effect of the current month (t) showed a positive effect of 0.11 (95% CI: −0.10–0.32), and the temperature effect of the following month (t + 1) exhibited a negative effect of −0.02 (95% CI: −0.22–0.18), suggesting that the impact of temperature on the death rate of police violence is primarily concentrated in the current month, without those significant advance or lag effects. Similar to previous studies [32,34], we integrated the temperature effects of the current month and the previous month as the overall effect to account for the displacement effects of temperature, and their net effect was still positive, indicating that the promoting effect of the temperature of the current month on police violence is greater than the possible inhibitory effect of the previous month. We also conducted the analysis to examine the effects of high temperature across different racial groups, which allowed us to explore the potential differences in temperature-related mortality risk among the victims of different races. The risk of police violence for different racial groups exposed to high temperatures is shown in S2 Fig, where the black race was found to be at a higher risk of police violence, while the white and Hispanic races were at a lower risk, and the Asian race had the lowest risk. This result is consistent with several previous studies on the statistics and analysis of police violence in the United States [16,21,25].

**Table 1:** The quantitative temperature effect on the death rate of police violence across different subgroups.

Subgroup	Temperature effect (%)	Standard error	P value	95% CI Lower	95% CI Upper
Above average Temperature	1.9485586	0.840	<0.05	0.3147079	3.5824093
Below average Temperature	2.2307894	0.636	<0.05	0.9854141	3.4761647
Above average Precipitation	0.4151138	0.499	>0.05	−0.55387	1.3840976
Below average Precipitation	1.2078494	0.334	<0.05	0.5420005	1.8736983
Above average Population	0.7982152	0.656	>0.05	−0.4915801	2.0880106
Below average Population	0.9086957	0.298	<0.05	0.3248021	1.4925894
Temperature: > 20℃	4.0228422	2.078	<0.05	−0.0510403	8.0967247
Temperature:10℃ ~ 20℃	−0.4619577	1.262	>0.05	−2.933708	2.0097925
Temperature: < 10℃	3.2921548	0.843	<0.05	1.6378155	4.9464941
Precipitation: > 100mm	0.3916145	0.456	>0.05	−0.5010606	1.2842897
Precipitation: 50 ~ 100 mm	0.4820731	0.373	>0.05	−0.2523284	1.2164746
Precipitation: < 50mm	2.0596748	0.580	<0.05	0.9226493	3.1967003
Population: > 5M	2.0036907	0.472	<0.05	1.0757213	2.9316601
Population: 1M ~ 5M	1.3396432	0.197	<0.05	0.9525707	1.7267158
Population: < 1M	0.8534159	0.275	<0.05	0.3145108	1.3923211
Month: t-1	−0.0173627	0.104	>0.05	−0.2213081	0.1865826
Month: t	0.1145677	0.108	>0.05	−0.1002016	0.329337
Month: t + 1	−0.0206721	0.104	>0.05	−0.2235865	0.1822422

### 3.3 Temperature effects on police violence risk over time and space

**Fig 3:**
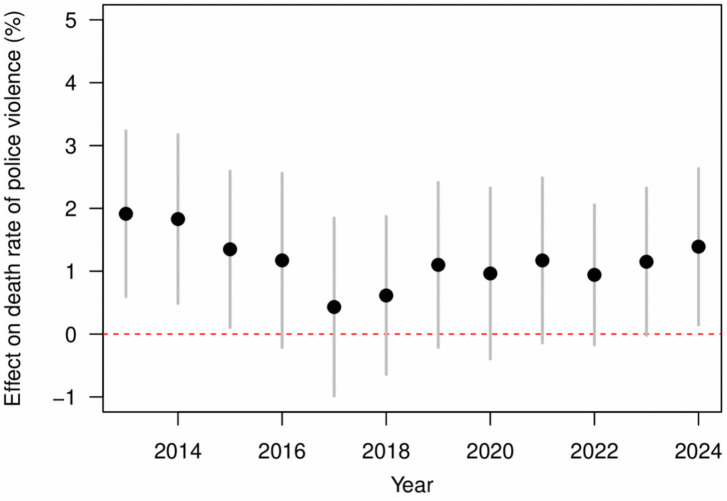
The effect of temperature on the death rate of police violence varies over different years. The black dots represent the effect of temperature for a specific year, with the 95% confidence interval represented by the grey lines. The red horizontal dashed line indicates the average temperature effect over the entire time period, estimated based on all samples.

**Fig 4:**
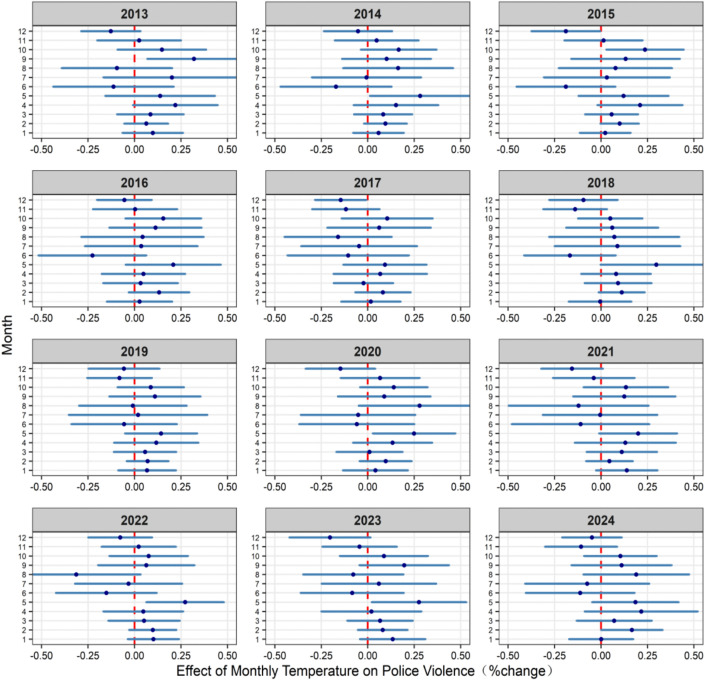
The effect of temperature on the death rate of police violence varies over different months. The blue dots represent the effect of temperature for a specific month, with the 95% confidence interval represented by the blue lines.

We interact the terms of temperature and precipitation with the state/climate region and year/month dummy variables, and calculate the interaction coefficients for specific state/climate region or year/month to explore the respective quantitative effects of temperature variation in the temporal and spatial dimensions in the continental United States (see Methods). The temperature effect was estimated as the increase in the death rate of police violence for each 1°C rise in temperature. Fig 3 and Table 2 demonstrate the estimated effect of temperature on the death rate of police violence over the time span from 2013 to 2024. The overall association between temperature and police violence exhibited a positive trend, indicating that the risk of police violence increased progressively with rising temperatures throughout the study period. The risk reached its highest level in 2013, declined gradually between 2013 and 2017, and subsequently demonstrated an upward trajectory in recent years. This persistent positive pattern underscores the potential role of temperature elevation as a continuous amplifying factor in the occurrence of police violence. It is noteworthy that the temperature-related effect of 1.39% (95%CI: 0.15–2.63) in 2024 was markedly higher than the variation observed over the preceding seven years (2017–2023), which may reflect the influence of exceptional circumstances such as extreme heat events or specific social dynamics that contributed to greater fluctuations relative to earlier periods. Fig 4 shows the temperature effect on the death rate of police violence in different months for a specific year, where higher temperatures in that month are confirmed to correspond to greater death rate of police violence. Overall, the magnitude of temperature effects varies across months within the time span of the study.

**Table 2:** The quantitative temperature effect on the death rate of police violence over different years.

Year	Temperature effect (%)	Standard error	95% CI Lower	95% CI Upper
2013	1.9141108	0.6732594	0.5945224	3.2336992
2014	1.8302027	0.6857317	0.4861686	3.1742368
2015	1.3494438	0.6356594	0.1035514	2.5953361
2016	1.1725046	0.7075181	−0.2142309	2.5592402
2017	0.4303738	0.7231032	−0.9869084	1.8476560
2018	0.6138442	0.6415699	−0.6436328	1.8713212
2019	1.1013301	0.6699872	−0.2118449	2.4145052
2020	0.9642591	0.6946944	−0.3973420	2.3258602
2021	1.1721822	0.6699736	−0.1409661	2.4853304
2022	0.9422001	0.5683374	−0.1717413	2.0561416
2023	1.1509669	0.5979487	−0.0210125	2.3229463
2024	1.3909359	0.6345853	0.1471488	2.6347229

**Fig 5:**
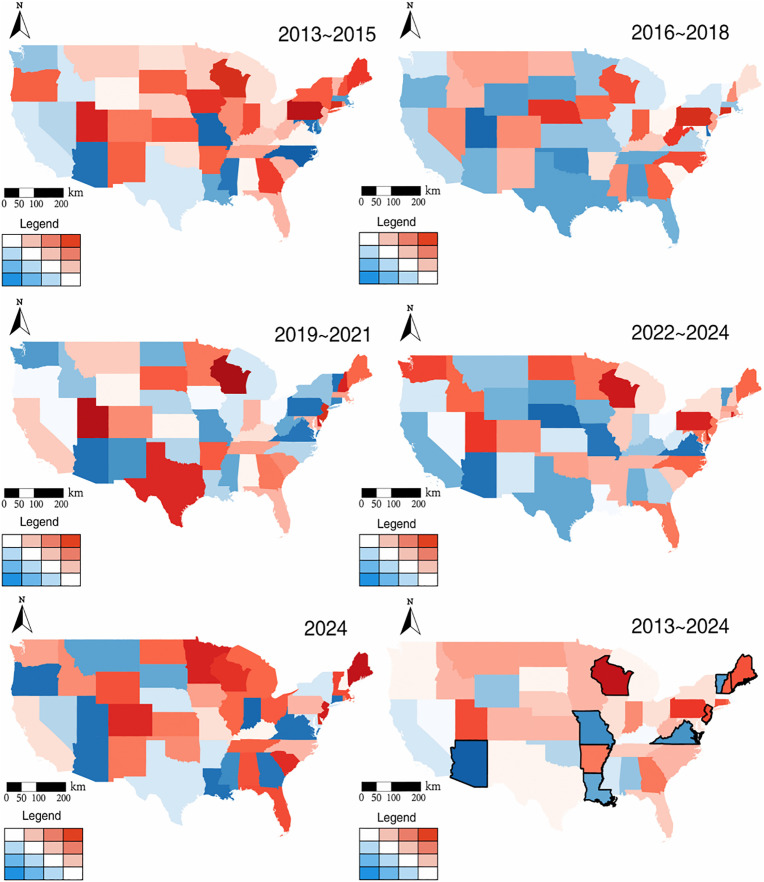
The effect of temperature on the death rate of police violence varies over different states. The color gradient approaching red indicates a stronger positive effect of temperature, whereas the gradient approaching blue reflects a stronger negative effect, with the black lines outlining states where the estimated effect is more pronounced relative to the rest of the country.

**Fig 6:**
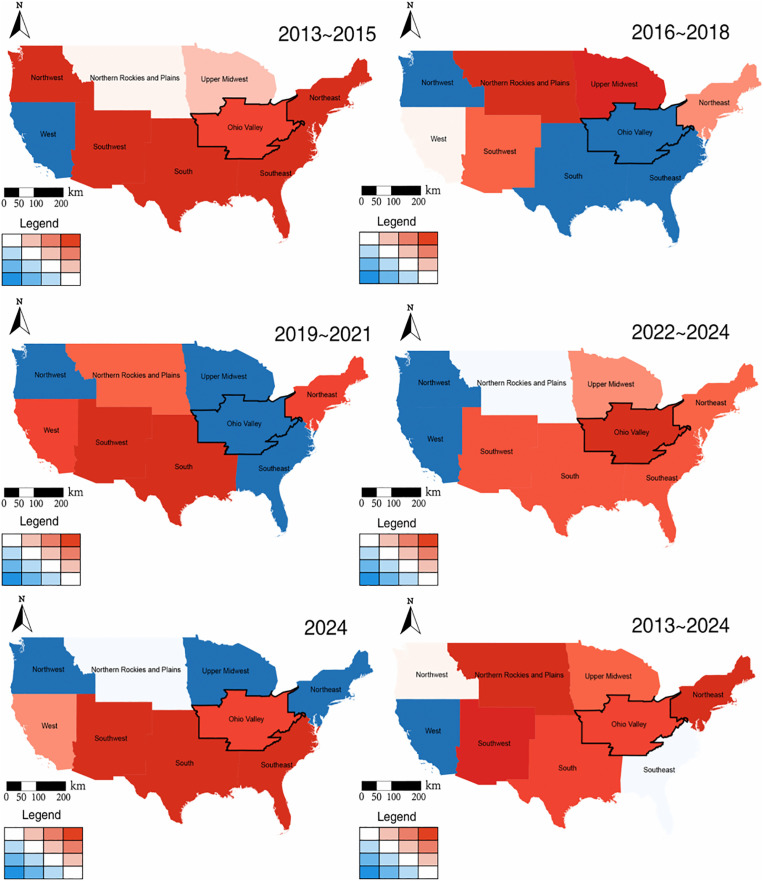
The effect of temperature on the death rate of police violence varies over different climate regions. The color gradient approaching red indicates a stronger positive effect of temperature, whereas the gradient approaching blue reflects a stronger negative effect, with the black lines outlining climate regions where the estimated effect is more pronounced relative to the rest of the country.

Figs 5 and 6 highlights the spatial variation in the impact of temperature on the death rate of police violence in the continental United States, revealing significant regional differentiations in the sensitivity to temperature variations. We found a positive association between temperature and police violence in numerous states and climate regions (shown in red), whereas a few states demonstrated negligible or slightly negative effects (shown in blue). For the states and climate regions with cooler baseline climates, each 1°C rise in temperature corresponds to a larger increase in the risk of police violence, while those with warmer climates are relatively moderate. For example, parts of the state involving Wisconsin, Maine and its areas to the north exhibits a higher relative risk, and traditionally hot areas including Texas, Arizona, and the Southeast and Southwest have more moderate temperature effects. The state-level effect of temperature is significant in Wisconsin, Maine and Pennsylvania, but less in some states, such as Texas, New Mexico and its adjacent southern regions. Across different climate zones, we found that certain areas in the West and Northwest were generally subject to relatively minor temperature effects, whereas regions in the Southwest and Northeast experienced more pronounced temperature impacts during specific periods. These findings highlight the spatial differentiation in the influence of elevated temperatures on the risk of police violence in the United States.

### 3.4 Effects of future expected temperature changes on police violence risk

**Fig 7:**
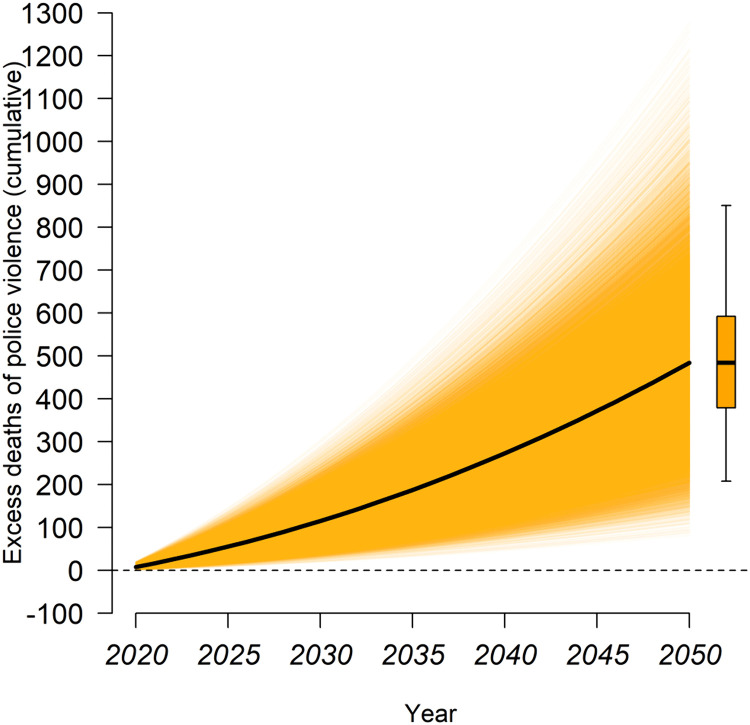
Cumulative excess deaths of police violence due to expected temperature changes in the United States by 2050 under the SSP5-8.5 scenario. The black line represents the median projection, and the yellow colored area shows the distribution of 30,000 Monte Carlo projections derived from resampled parameter estimates and 30 climate models. The box plot illustrates the median, interquartile range, and 95% confidence interval for the estimated cumulative excess deaths projected through the year 2050.

Utilizing the integration of shared socioeconomic pathways and typical concentration trajectories from the International Coupled Model Comparison Program Phase 6 (CMIP6) [38], we projected the changes in the mean annual temperature across the continental United States under the highest greenhouse gas emissions scenario of SSP5–8.5. Incorporating the anticipated population growth trends [40], we further estimated the additional excess deaths resulting from police violence attributable to the expected temperature rise between 2020 and 2050 (see Methods). Fig 7 illustrates the projected cumulative excess deaths of temperature increases by the year 2050 under the SSP5–8.5 scenario. The results indicate that, relative to the year of 2020, the estimated additional cumulative deaths attributable to police violence exacerbated by high temperatures are projected to reach 114 (95% CI: 28–229), 263 (95% CI: 84–473), and 479 (95% CI: 183–836) by 2030, 2040, and 2050, respectively. The lower limit of the additional cumulative deaths indicates that adaptive strategies or behavioral changes may reduce the risk, while the upper limit indicates that the risk of police violence will increase under the situation of unmitigated warming and increased social vulnerability. These projections reflect the multifaceted and long-term implications of climate change on public safety, suggesting that sustained increases in temperature may amplify the frequency of police violence incidents through both direct physiological stress and indirect socio-environmental pathways.

### 3.5 Robustness analysis

To ensure the robustness of our findings with respect to model specification and data selection, we conducted several sensitivity tests. First, we estimated the main model using alternative regression methods with different fitting functions and specifications, such as natural cubic spline with varying degrees of freedom, higher-order polynomial, and B-spline. The resulting curves remained largely consistent (see Results), suggesting that the choice of functional form did not influence the outcomes. Second, we expanded the timeframe from the current one month to previous or following two months to assess whether we accounted for the full duration of the displacement effect, and the temperature effect on the death rate of police violence within each range was quantified, respectively. These findings, presented in S3 Fig, indicate that using the sum of the temperature effects of the current month and the previous month as the overall effect fully reflects the displacement effect of temperature [32,34], and validate the robustness of our results. Additionally, we tested various combinations of confounding factors, including the precipitation and the variables of state-year and county-month, with the results shown in S1 Table. The results indicate that adjustment or modification of the above factors exert less influence on the temperature effect with respect to the death rate of police violence, which further supports the reliability of our study.

## 4. Discussion

The global climate is changing at an unprecedented rate, especially with rising temperatures [3,5]. Exposure to heat due to rising temperatures contributes to an increased risk for several health-related and public safety events [6,8,9], including various types of violence or crime [16,18]. In this study, we demonstrate that higher temperatures are associated with increased risk for police violence in the United States, in which several potential mechanisms account for these findings.

The exposure-response curve demonstrates an overall increasing trend between ambient temperatures and the death rate of police violence, which is consistent with the results of other studies involving temperature and gun violence or crime [14,18,22]. The results indicate that higher temperatures are associated with increased risk of police violence, which may be related to the specific nature of the interaction between police and the victims under environmental heat stress. The emergence of higher temperature exposure as an important risk factor for police violence is consistent with the theory that heat stimulates aggression or violence [43,44]. Research has shown that high temperatures can influence violence and crime by altering daily activities, behavioral patterns, and physiological responses [45,46]. Warmer conditions encourage outdoor activities, while heat affects serotonin regulation, which can heighten impulsivity and intensify social interactions—factors that contribute to a greater risk of conflict and violent incidents [18]. Extreme temperatures can also impact drinking habits, induce emotional instability, and increase vulnerability to victimization [46,47]. Excessive heat causes physical discomfort and psychological distress, which in turn elevate aggression. As heat stress intensifies, individuals experience heightened discomfort, fostering irritability and increasing the likelihood of aggressive behavior and criminal actions [48]. Furthermore, higher temperatures can undermine psychological stability, amplify negative emotions, and reduce self-control, all of which contribute to a rise in violent tendencies [49]. Excess heat exposure has been linked to increased anger and hostility, diminished vigilance, and heightened aggression [50]. Environmental heat stress can also exacerbate psychological discomfort and stress, ultimately raising the risk of criminal behavior [51]. Individuals who endure greater physiological heat stress tend to be more aggressive and more prone to violent reactions [52]. Higher temperatures can amplify aggressive tendencies beyond one’s usual temperament, escalating conflicts [53]. Additionally, heightened hostility and physiological arousal due to heat exposure further fuel aggression. Prolonged exposure to high temperatures may also alter decision-making strategies, weaken future-oriented thinking, and reduce self-regulation—factors that collectively increase the likelihood of violent behavior [54]. Sustained high temperatures can lead to emotional irritability among residents, which has a large negative impact, and involves multiple factors such as race, poverty, and law enforcement policies [18]. Higher temperatures can amplify these potential conflicts and eventually erupt in interactions with the police [14,25]. These dynamics help explain why the risk of police violence and related fatalities tends to rise following periods of higher heat exposure.

Our results suggest that the risk of police violence does not increase in a strictly linear trend with temperature, but rather appears linear across both high and low temperature ranges. This nonlinearity may reflect the behavioral thresholds induced by heat, affecting both victims and police officers, increasing the likelihood of interactive conflict [16,22]. The analysis indicates that given the time scale limitations of monthly data, the promoting effect of high temperature on police violence is still pronounced, which is consistent with previous results on monthly temperature and health-related and public events [32,55]. We found that the impact of temperature on the death rate of police violence in 2024 was higher than in previous years. Considering that 2024 is a presidential election year with fierce competition between the major parties, the Democratic and Republican parties, and that 2024 is the hottest year on record, with the global average temperature exceeding 1.5°C for the first time [56,57], the United States may experience more frequent and intense heat waves. In the high-pressure environment of an election year, social factors such as political polarization and tensions (election-related unrest, public distrust of the police, and social policy changes) may be superimposed on higher temperatures, resulting in a larger effect for the risk of police violence [16,44]. At the same time, higher temperatures act as an amplifier of latent social tensions, which increase the incident of conflict in volatile environments [15,28]. Our research projects that by the year 2050, the cumulative additional deaths from police violence in the United States due to expected temperature change would achieve a substantial increase. Given the profound and widespread societal impact of the event of deaths of police violence, this phenomenon could inform public health strategies, including police training for heat waves or community cooling measures, to mitigate the risks posed by future temperature rises. Our findings indicate a positive association between rising temperatures and an increased risk of police violence. It is important to emphasize that this relationship should be interpreted as an association rather than a causal link [22]. The analysis was not designed to establish causality, and no direct causal evidence can be inferred from the present study. High temperatures can increase the aggressive tendencies in both civilians and law enforcement officers, which may heighten the likelihood of violent encounters [16]. Heat exposure may also impair officers’ emotional regulation, patience, and risk perception, thereby increasing the risk of violent responses during police operations [18]. In addition, variation across policing contexts may play a role. Officers on foot patrol are more likely to experience the physiological and psychological burden of heat, while officers in vehicles may be partially protected by air conditioning, which can reduce the adverse effects of high temperatures [22,32]. Future research should investigate these potential mechanisms using individual-level behavioral data and occupation-specific analyses in order to clarify how heat influences the dynamics of police–civilian interactions. Consistent with prior researches [16,18], our findings suggest that the association between temperature and violence may be context dependent. Notably, a meta-analysis of laboratory studies was conducted [58], where no general effect of temperature on antisocial or violent outcomes were demonstrated. This contrast between controlled experimental settings and population-level observations underscores the potential role of broader social and structural factors in shaping the observed relationship. In the present study, we utilized measured temperature as the primary predictor to examine its association with police violence. This approach enables the characterization of effects across varying magnitudes of temperature exposure, which may involve complex nonlinear relationships. Specifically, the response patterns at extreme high and low temperatures are not necessarily symmetrical, and capturing these variations provides critical insight into the potential influence of temperature on violent outcomes. Although both measured temperature [7,23,32] and temperature deviation [16,55,59] from a long-term reference period are widely employed in the literature, there is currently no universally accepted standard. The examination of temperature anomalies represents a valuable complementary strategy, and future research will consider this approach to further investigate the relationship between deviations from normal temperature and the risk of police violence. Additionally, the risk of police violence for the black race was found to be at a higher risk of police violence, while the white and Hispanic races were at a lower risk, and the Asian race had the lowest risk. The racial category of “unknown” had the highest risk. This result is consistent with several previous studies on the statistics and analysis of police violence in the United States [16,21,25], which indicates that the transparency and compliance of police enforcement still need to be improved, suggesting that racial identity remains a significant factor influencing the violent incidents in American society [16].

There are some limitations in this study. The behavioral variations of both police officers and victims are influenced by a range of complex factors beyond environmental conditions [14]. Individual-level variables such as gender, adaptability, attitude, and health status can shape physiological responses and behavioral patterns during conflicts under heat stress [15]. However, due to the challenges of environmental complexity and monitoring limitations, these factors cannot be precisely measured or controlled as the variables, introducing the potential bias in quantifying the temperature effects. Among the factors potentially influencing the vulnerability of victims, socioeconomic status (SES) is likely to play an important role [16,20]. Individuals with lower SES may have reduced access to resources such as adequate housing, cooling or heating, and healthcare, which could increase susceptibility to extreme temperatures [60]. Incorporating measures of SES and exploring potential interaction effects with temperature could provide a more nuanced understanding of the social determinants underlying vulnerability to temperature-related risk. Investigating whether SES modifies the association between temperature and police violence represents an important avenue for future research. The analysis does not account for the use of cooling measures like air conditioning by those involved in the conflicting parties due to the data limitations, which may lead to an overestimation of the assessed effect of temperature [22]. Although precipitation was treated as a confounding factor in our analysis, we acknowledge that it may also exert an independent or moderating influence on police violence. Previous studies suggest that precipitation can affect mortality [61,62], suicide [32,63], and other public safety or violent incidents [60,64,65], indicating its potential relevance to our topic. Since the primary focus of our study is the impact of temperature exposure on police violence, a separate analysis of the independent effects of precipitation was not conducted in this study. Future research could further investigate the potential moderating or independent role of precipitation, as this relationship provides a more comprehensive understanding of how different weather conditions shape the occurrence of police violence.

Police violence is a significant social security concern that endangers both individuals and communities, posing a major public health challenge. The impact of higher-temperature environments on the risk of police violence should not be overlooked. Individuals prone to aggressive behavior are advised to minimize exposure to extreme heat whenever possible. Under the context of rapid climate change and increasingly frequent extreme weather events, it is crucial to implement effective oversight and management strategies for law enforcement of police. Additionally, our study provides a comprehensive understanding of the relationship between ambient temperature and police violence, offering the valuable insights into this critical issue of society.

## 5. Conclusion

In this study, we focused on the scope of violent incidents to the special and urgent area of police violence, and found that rising temperatures were significantly positively correlated with the death rate of police violence. The results suggest that the additional deaths of police violence due to the expected temperature rise of climate change poses an urgent challenge to public health, and targeted public health intervention strategies are recommended under specific high temperature environmental conditions, especially during the social tensions such as election years, to avoid extreme climate events triggering large-scale social instability.

## Supporting information

S1 Fig Spatial distribution of police violence-related deaths. (TIF)

S2 Fig The risk of police violence for different racial groups exposed to high temperatures. (TIF)

S3 Fig The displacement effects of previous and following two months of temperature on the death rate of police violence. (TIF)

S1 Table The effects of temperature on the death rate of police violence with the control of different combinations of the interference factors in the model. (DOCX)
