# Supplementary figures and images for: Higher temperatures are associated with increased risk of police violence: A nationwide county-level study in the United States, 2013–2024
Source: PLoS One. 2026 Mar 20;21(3):e0345523. doi: 10.1371/journal.pone.0345523 (PMC13004362; doi:10.1371/journal.pone.0345523)

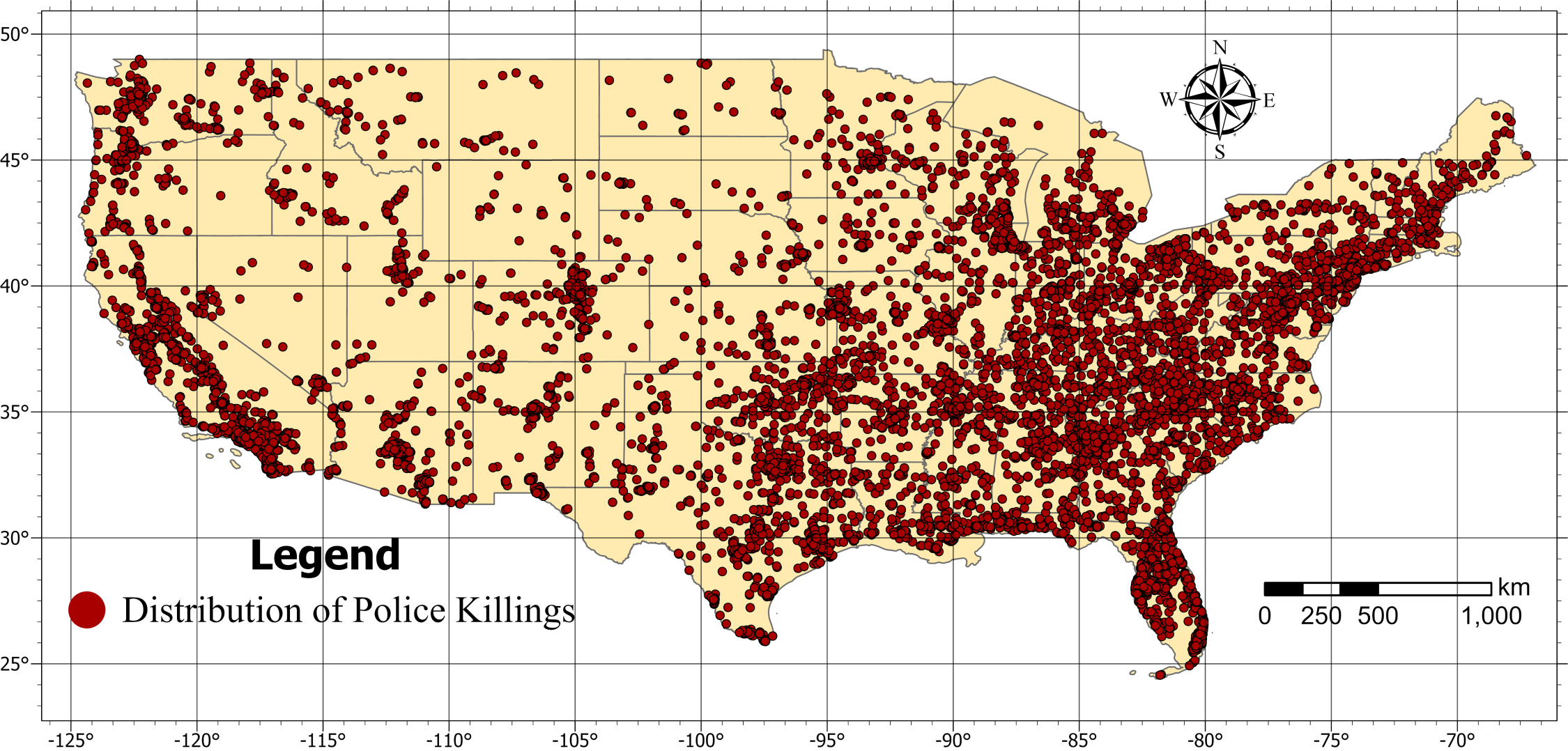

Supplement: S1 Fig — (TIF) [file pone.0345523.s001.tif]
