# Supplementary material for: Higher temperatures are associated with increased risk of police violence: A nationwide county-level study in the United States, 2013–2024
Source: PLoS One. 2026 Mar 20;21(3):e0345523. doi: 10.1371/journal.pone.0345523 (PMC13004362; doi:10.1371/journal.pone.0345523)
Supplement: S1 Table — (DOCX) [file pone.0345523.s004.docx]

**S1 Table. The effects of temperature on the death rate of police violence with the control of different combinations of the interference factors in the model.**

|  | Change in death rate of police violence for each 1°C rise (%) | | | | | |
| --- | --- | --- | --- | --- | --- | --- |
| Confounder choice | Less than 50mm precipitation | | | Larger than 5 million population | | |
|  | Estimated effects and 95% CI | | | Estimated effects and 95% CI | | |
| county-month, state-year, precipitation | 2.04 | 0.91 | 3.21 | 2.02 | 1.09 | 2.94 |
| county-month, state-year, | 2.07 | 0.92 | 3,22 | 2.03 | 1.10 | 2.95 |
| county-month, precipitation | 2.03 | 0.90 | 3.19 | 2.01 | 1.08 | 2.93 |
| state-year, precipitation | 2.05 | 0.89 | 3.18 | 2.04 | 1.11 | 2.96 |
